# The increased risk of exposure to fine particulate matter for depression incidence is mediated by elevated TNF-R1: the Healthy Aging Longitudinal Study

**DOI:** 10.1265/ehpm.25-00106

**Published:** 2025-06-27

**Authors:** Ta-Yuan Chang, Ting-Yu Zhuang, Yun-Chieh Yang, Chih-Cheng Hsu, Wan-Ju Cheng

**Affiliations:** 1 Department of Occupational Safety and Health, College of Public Health, China Medical University, No. 100, Sec. 1, Jingmao Rd., Beitun Dist., Taichung City 406040, Taiwan; 2 National Center for Geriatrics and Welfare Research, National Health Research Institutes, No. 35, Keyan Rd., Miaoli County 350401, Taiwan; 3 Institute of Population Health Sciences, National Health Research Institutes, No. 35, Keyan Rd., Miaoli County 350401, Taiwan; 4 Department of Psychiatry, China Medical University Hospital, No. 2, Yude Rd., North Dist., Taichung City 404327, Taiwan; 5 Department of Public Health, China Medical University, No. 100, Sec. 1, Jingmao Rd., Beitun Dist., Taichung City 406040, Taiwan

**Keywords:** Air pollution, Inflammation, Mental health, Particulate matter, Road traffic noise

## Abstract

**Background:**

Depression among older adults is an important public health issue, and air and noise pollution have been found to contribute to exacerbation of depressive symptoms. This study examined the association of exposure to air and noise pollutants with clinically-newly-diagnosed depressive disorder. The mediating role of individual pro-inflammatory markers was explored.

**Methods:**

We linked National Health Insurance claim data with 2998 healthy community-dwellers aged 55 and above who participated in the Healthy Aging Longitudinal Study between 2009 and 2013. Newly diagnosed depressive disorder was identified using diagnostic codes from the medical claim data. Pollutants were estimated using nationwide land use regression, including PM_2.5_ and PM_10_, carbon monoxide, ozone, nitrogen dioxide, sulfur dioxide, and road traffic noise. Cox proportional hazard models were employed to examine the association between pollutants and newly developed depressive disorders. The mediating effect of serum pro-inflammatory biomarkers on the relationship was examined.

**Results:**

Among the 2998 participants, 209 had newly diagnosed depressive disorders. In adjusted Cox proportional hazard models, one interquartile range increase in PM_2.5_ (8.53 µg/m^3^) was associated with a 17.5% increased hazard of developing depressive disorders. Other air pollutants and road traffic noise were not linearly associated with depressive disorder incidence. Levels of serum tumor necrosis factor receptor 1 mediated the relationship between PM_2.5_ and survival time to newly onset depressive disorder.

**Conclusion:**

PM_2.5_ is related to an increased risk of newly developed depressive disorder among middle-aged and older adults, and the association is partially mediated by the pro-inflammatory marker TNF-R1.

**Supplementary information:**

The online version contains supplementary material available at https://doi.org/10.1265/ehpm.25-00106.

## 1. Background

Depression accounts for 4.3% of the global burden of disease and 11% of all years lived with disability [1]. In the older population, depression is prevalent due to increased risk factors including medical illnesses, cognitive impairment, and disability. Reciprocally, depression further worsens health and promotes disability [2].

Air pollution has been found to be a modifiable risk factor of depression [3]. However, which specific air pollutant is related to depression remains controversial. For example, although the evidence for particulate matter (PM) with an increased risk of new onset depressive symptoms was the most robust among various pollutants, there were still inconsistent findings [4–7]. Other pollutants including SO_2_, NO_2_, and CO have been shown to increase the risk of depression [7, 8], but study heterogeneity was high [9]. The association between air pollutants and depression and depressive symptoms in older adults was conflicting as well [10, 11]. A major limitation for studies concerning environmental pollution and depression is the use of self-reported questionnaires to identify depression and depressive symptom exacerbation. Questionnaires capture short-term mood state, which is easily influenced by stressful events, rather than reflecting sustained neurological function change as observed in depressive disorders.

The mechanisms of air pollutants’ effect on mood have been thought to be neuro-inflammation, oxidative stress [12], and stress hormone production [13]. Animal studies showed that air pollutants increased pro-inflammatory factors, such as interleukin-6 (IL-6), tumor necrosis factor-alpha (TNF-alpha), nuclear factor kappa-light-chain-enhancer of activated B cells (NF-kB), and induced neurotoxicity [14, 15]. Air pollutants may also affect mental health indirectly through cerebrovascular damage and neurodegeneration [16, 17]. In addition, air pollution was linked to cardiovascular disease, cerebrovascular diseases, lung disease, and cancer, which are major risk factors for depression in older adults [17–20].

Air pollution and noise pollution are closely intertwined in urban areas with heavy traffic, and both can affect the nervous system through mechanisms involving systemic inflammation [21]. Noise pollution has been suggested to be associated with depression, but the evidence has been inconsistent. Two meta-analyses observed a significant but small effect of aircraft noise, and no effect of road traffic noise, on depression [22, 23]. Longitudinal studies are scarce, and one in Germany observed that noise annoyance predicted new-onset symptoms of depression and anxiety [24]. Another longitudinal study found an effect of transportation noise on depression was mediated by noise annoyance [25]. One community-based study reported an association between road traffic noise exposure with an increased prevalence of depression, particularly at 1,000 and 2,000 Hz [26]. Neuroinflammation has been proposed to be the mechanism of noise leading to depression in an animal study [27].

Studies focusing air and noise pollutants and depression in the older population remain scarce, but the topic shows importance in promoting mental health in this population. The current study aims to fill the knowledge gap concerning the impact of environmental pollutant exposure on newly diagnosed depression by (1) adopting a longitudinal design and examining the association between pollutant exposure and depression incidence, and (2) examining the role of individual inflammatory markers and physical illnesses in mediating the association.

## 2. Methods

### 2.1 Participants

We used data from the Healthy Aging Longitudinal Study in Taiwan (HALST) [28]. The HALST recruited healthy community-dwellers aged 55 and above in Taiwan. The first wave survey was conducted in 2009–2013, and the second wave follow-up in 2014–2019. A total of 5663 participants completed the first wave survey. Participants completed the consent form and were interviewed at home. Within two weeks, they received a physical examination and laboratory blood tests. This study was approved by the Institutional Review Board of the National Health Research Institutes (EC1120201-E).

**Fig. 1:**
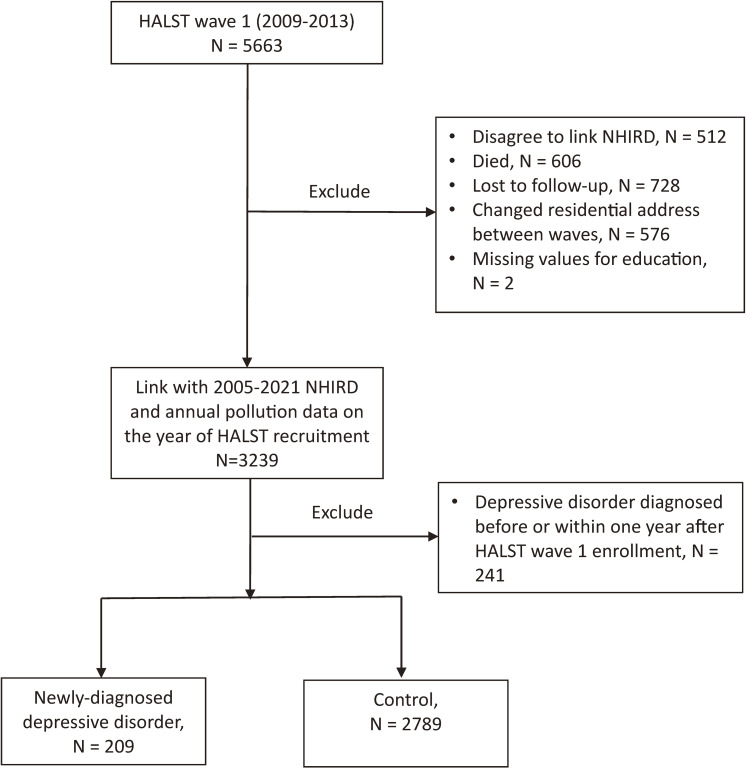
Sample-selection flowchart

We then linked HALST data with the National Health Insurance Research Database (NHIRD); however, 512 participants did not consent to data linkage and were excluded from analysis. To confirm location-based pollution exposure, we retrieved participants’ household addresses. Only those who completed both waves of the HALST survey and maintained the same residential address were included to ensure consistent pollutant exposure over time. Between the first and second waves, we excluded 606 participants who had died, 728 lost to follow-up, 576 who changed their address, and 2 with missing education data (Fig. 1). To avoid protopathic bias, participants who were identified as having depressive disorder in the NHIRD before HALST wave 1 or within one year after enrollment in HALST wave 1 were excluded as well. Finally, 2998 participants were followed for depressive incidence until the occurrence of depressive disorders, death, or December 2021, whichever came first.

### 2.2 Environmental pollution measurements

Exposure assessment for each participant is estimated by nationwide land use regression models [29], including 24-h average day-evening night noise level (Lden) in A-weighted decibels (dBA), ozone (O_3_), CO, SO_2_, NO_2_, PM_10_, and PM_2.5_. Data of road traffic noise and air pollutants were collected from 151 noise and 74 air quality monitoring stations over the whole island. The methods to build prediction models of the nationwide land use regression followed the steps mentioned in past studies [30–32]. The model performance of R^2^ for noise and air pollutants built in 2019 ranged from 0.44 (Lden) to 0.84 (CO). Exposure variance was adjusted by the difference between the year of HALST wave 1 recruitment and 2019 based on the annual average in the EPA monitoring station closest to subjects’ residential addresses. Based on the above models and time adjustment, we estimated the annual mean exposure value by each participant’s residential address in the year of HALST wave 1 recruitment.

### 2.3 Depressive disorder

Depressive disorder was identified using the NHIRD data from January 2005 to December 2021. Taiwan’s universal National Health Insurance program was launched in 1995 and covers approximately 99% of the national population [33]. The NHIRD was made available by the Health and Welfare Data Science Center. Before 2016, depressive disorders were defined using the International Classification of Diseases, 9th Revision Clinical Modification (ICD-9-CM), with ICD-9 codes 296.2, 296.82, 300.4, and 311. Starting from 2016, the International Classification of Diseases, 10th Revision Clinical Modification (ICD-10-CM) has been utilized for this database, with ICD-10 codes F32, F33, and F34.1 being used to identify depressive disorders. At least two outpatient visits or at least one hospitalization claim of depression was required for identification as having a depressive disorder [34, 35]. The index date is the date of the first inpatient or outpatient record for depressive disorders.

### 2.4 Inflammatory markers and anthropometric variables

Five serum inflammatory markers were included: tumor necrosis factor receptor 1 (TNF-R1), neutrophil-to-lymphocyte ratio (N/L), high sensitivity C-reactive protein (hs-CRP), insulin-like growth factor 1 (IGF-1), and high sensitivity interleukin-6 (IL-6HS). Body weight and height were obtained during clinical examination, and body-mass index (BMI) was calculated as body weight (kg) divided by the square of body height (m). These variables were collected from HALST wave 1. Missing values were imputed using mean values.

### 2.5 Physical illnesses

Heart diseases, cerebrovascular diseases, and dementia potentially mediated the relationship between pollutants and depressive disorders. They were identified if there were two outpatient records or one hospitalization record between one year after exposure to pollutants and the end of follow-up. Heart diseases include myocardial Infarction (ICD-9 codes 410, 412 or ICD-10 codes I21, I22, I25.2) and heart failure (ICD-9 codes 428 or ICD-10 codes I09.9, I11.0, I13.0, I13.2, I25.5, I42.0, I42.5, I42.8–I42.9, I43). Cerebrovascular diseases include cerebral hemorrhage, infarction, and artery diseases (ICD-9 codes 430–437 or ICD-10 codes G45, G46, I60–I68). Dementia was identified by ICD-9 codes 290, 331.0, 331.2 or ICD-10 codes F00–F03, F05.1, G30.

Besides the above diagnosis, the Charlson Comorbidity Index (CCI) was used to evaluate physical comorbidity burden and was adjusted in survival models. The CCI is the sum of the weighted scores of 19 comorbid conditions [36]. In this study, CCI was calculated with 15 physical conditions, excluding myocardial infarction, heart failure, cardiovascular disease, and dementia. Participants were categorized into 3 groups by CCI score 0, 1, and ≥2.

### 2.6 Sociodemographic variables and health behaviors

Age, sex, education, marital status, work status, family income, smoking, and exercise behaviors were self-reported from the HALST. Education level was categorized into two groups: primary education or less and secondary education or above. Marital status was dichotomized into married and other statuses (separated, divorced, widowed, or single). Participants reported whether they were currently working or not. Participants provided information on household income by selecting from seven predefined categories. However, 43.1% of participants either left this information as unknown or chose not to disclose it. To address this, a dummy variable was created to account for the missing household income data. Participants were further categorized into high- and low-income groups, using a threshold of 50,000 New Taiwan dollars (approximately US$1,542) per month.

Regarding health behaviors, smoking status was assessed through participant self-reporting of current smoking habits, categorized as either yes or no. Exercise habits were assessed by asking participants if they had engaged in any form of exercise over the past year, including but not limited to running, boxing, or dancing. Metabolic equivalents (METs) were calculated based on subjective report of overall physical activity from the HALST [37].

### 2.7 Statistical analysis

Descriptive statistics were conducted using Chi-square tests and Mann-Whitney tests to compare sociodemographic characteristics, inflammatory markers, physical illnesses, and exposed levels of pollutants between participants who developed depressive disorder and the control group. The association between pollutants and depressive disorders was examined using Cox proportional hazard models with restricted cubic splines to account for non-linear relationships [38]. Three knots were set at the 10th, 50th, and 90th percentiles. Follow-up was censored by death or December 2021, whichever came first. The Cox models were adjusted for age, gender, BMI, education level, marital status, work status, family income, cigarette smoking, exercise habits, METs, and CCI. We examined the hazard ratio for depression associated with a one-interquartile range (IQR) increase in pollutant levels. The regression analysis was also conducted using pollutant levels categorized by quartiles. Data analysis was performed using SAS version 9.4 (SAS Institute).

We used causal mediation analysis to evaluate the relationship between pollutants and depressive disorder with the addition of inflammatory markers and physical illnesses (i.e., heart diseases, cerebrovascular diseases, and dementia) to the model. Inflammatory markers were log transformed for analysis. Confidence intervals (CIs) for mediation effect estimates were generated using bootstrapping with 500 simulations. Control variables included age, gender, BMI, education level, marital status, family income, cigarette smoking, exercise habits, METs, and CCI. Mediation analyses were conducted using the “mediation” package in R (v.4.4.0), with time to depressive disorder incidence as outcome modeled using a parametric survival regression model.

## 3. Results

Among the 2998 participants, 209 were identified as having newly diagnosed depressive disorders following pollution exposure. Compared to those without depressive disorders, participants diagnosed with depressive disorders were older, had a higher proportion of females, a lower proportion of smoking, a lower prevalence of heart disease and cerebrovascular disease, a higher prevalence of dementia, and a greater proportion with a CCI ≥ 1 (Table 1).

**Table 1:** Baseline demographic characteristics of study participants (N = 2998).

**Characteristic**	**Depression**	**Control**	***p^a^***
	**(n = 209)**	**(n = 2789)**	

	**N (%)**	**N (%)**	
Gender (male)	84 (40.19)	1382 (49.55)	0.011
Education (primary or lower)	105 (50.24)	1446 (51.85)	0.706
Marital status (married)	165 (78.95)	2130 (76.37)	0.445
Work status (working)	53 (25.36)	797 (28.58)	0.360
Family income			0.393
High	29 (13.88)	455 (16.31)	
Low	81 (38.76)	1140 (40.87)	
Missing	99 (47.37)	1194 (42.81)	
Cigarette smoking (yes)	11 (5.26)	354 (12.69)	0.002
Regular exercise (yes)	162 (77.51)	2049 (73.47)	0.230
CCI			0.001
0	44 (21.05)	950 (34.06)	
1	69 (33.01)	806 (28.90)	
≥2	96 (45.93)	1033 (37.04)	
Heart disease	12 (5.74)	409 (14.66)	0.001
Cerebrovascular disease	26 (12.44)	527 (18.90)	0.026
Dementia	40 (19.14)	275 (9.86)	<0.001


	**Mean (SD)**	**Mean (SD)**	***p^a^***
Age (year)	69.59 (7.33)	68.34 (7.71)	0.023
BMI (kg/m^2^)	24.45 (3.42)	24.71 (3.41)	0.247
METs	1679.12 (1977.54)	1851.11 (2828.18)	0.355
N/L ratio	2.04 (0.88)	1.98 (0.87)	0.260
hs-CRP (mg/dL)	0.21 (0.46)	0.21 (0.54)	0.767
IGF-1 (ng/mL)	69.72 (21.03)	70.62 (23.69)	0.785
TNF-R1 (pg/mL)	1192.61 (472.13)	1246.25 (680.21)	0.149
IL-6HS (pg/mL)	1.9 (1.78)	1.84 (1.64)	0.898

*Abbreviations:* METs = Metabolic equivalents; BMI = Body Mass Index; CCI = Charlson Comorbidity Index; TNF-R1 = tumor necrosis factor receptor 1; N/L ratio = neutrophil-to-lymphocyte ratio; hs-CRP = high sensitivity c-reactive protein; IGF-1 = insulin-like growth factor 1; IL-6HS = high sensitivity interleukin-6; SD = standard deviation.

^a^P values comparing the two groups were calculated using the Mann-Whitney test and chi-square tests.

Using Mann-Whitney tests, levels of air pollutants were not significantly different between participants with and without newly diagnosed depressive disorder (Table 2). The distribution of pollutants (mean, median, and IQR) of the study population can be found in supplementary Table S1. Noise was significantly and slightly lower in the depression group than in the control group (60.7 vs. 61.3 dBA).

**Table 2:** Annual exposure levels of air pollutants and road traffic noise.

**Variable**	**Depression**		**Control**		**p^a^**
	
	**(n = 209)**		**(n = 2789)**		
	
	**Median**	**IQR**	**Median**	**IQR**	
PM_2.5_ (µg/m^3^)	30.758	9.951	29.840	8.278	0.103
PM_10_ (µg/m^3^)	52.288	17.418	50.961	15.480	0.294
CO (ppb)	0.572	0.148	0.587	0.164	0.578
NO_2_ (ppb)	10.821	5.561	10.484	4.546	0.306
O_3_ (ppb)	27.907	9.763	28.281	10.262	0.979
SO_2_ (ppb)	4.208	1.593	3.864	1.688	0.098
L_den_ (dBA)	60.669	5.218	61.316	5.237	0.043

*Abbreviations:* dBA = A-weighted decibel.

^a^P values comparing between the two groups using the Mann-Whitney test.

In adjusted Cox proportional hazard models with restricted cubic splines, all pollutants showed linear relationship with incidence of depressive disorder, except for SO_2_ (Supplementary Fig. S1). An interquartile range (IQR) increase in PM_2.5_ (8.53 µg/m^3^) was associated with a 17.5% increased hazard of developing depressive disorders (adjusted hazard ratio [HR] = 1.175, 95% CI = 1.012–1.363; Table 3). Other pollutants and road traffic noise were not linearly associated with the incident risk of depressive disorder.

**Table 3:** Cox Proportional Hazards Model for Depressive Disorder Incidence.

**Pollutants (IQR)**	**Crude model^a^**		**Adjusted model^a^**	
	
	**HR (95% CI)**	**p**	**HR (95% CI)**	**p**
PM_2.5_ (8.53 µg/m^3^)	1.181 (1.020–1.367)	0.026	1.175 (1.012–1.363)	0.034
PM_10_ (15.81 µg/m^3^)	1.088 (0.912–1.297)	0.352	1.090 (0.912–1.302)	0.345
CO (0.16 ppb)	1.062 (0.939–1.200)	0.338	1.027 (0.908–1.162)	0.674
NO_2_ (4.60 ppb)	1.120 (0.955–1.313)	0.163	1.078 (0.917–1.268)	0.364
O_3_ (10.24 ppb)	0.966 (0.808–1.154)	0.701	1.035 (0.857–1.249)	0.724
SO_2_ (1.69 ppb)	1.051 (0.894–1.235)	0.549	1.043 (0.888–1.225)	0.610
L_den_ (5.3 dBA)	0.937 (0.839–1.046)	0.937	0.933 (0.834–1.044)	0.228

*Abbreviations:* dBA = A-weighted decibel;

^a^The hazard ratio (HR) and 95% confidence interval (CI) for the occurrence of depressive disorders were estimated for pollutants categorized into quartiles, with the lowest quartile serving as the reference group (N = 2,998). Crude models were univariate Cox proportional hazard models, showing the estimated increased hazard with an IQR increase in the pollutants. Adjusted models were controlled for age, gender, body mass index, education level, marital status, work status, family income, cigarette smoking, exercise behaviors, metabolic equivalents, and Charlson comorbidity index.

**Fig. 2:**
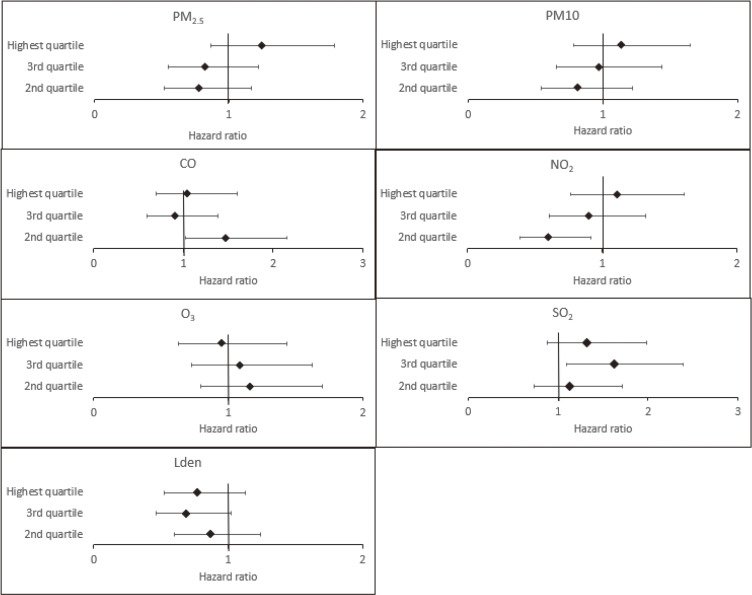
Association between quartiles of pollutants and the incidence of depressive disorder in Cox regression models. The lowest quartiles serve as the reference group.

We further categorized pollutants into quartiles (Fig. 2 and Supplementary Table S2). The hazard ratio for depression incidence was 1.5-folds higher in the second quartile of CO compared to the lowest quartile (adjusted HR = 1.480, 95% CI = 1.019–2.149). Similarly, the third quartile of SO_2_ was associated with a 1.6-folds higher risk than the lowest quartile (adjusted HR = 1.618, 95% CI = 1.096–2.390). The association between NO_2_ and the risk of depression was lowest in the second quartile. PM_2.5_ quartiles were not associated with depression in the cox regression model, but depression incidence was highest (9.1%) in the highest quartile of PM_2.5_ exposure and lowest in the second quartile (5.7%) (Supplementary Table S3). In Cox regression models, the highest quartile of PM_2.5_ was associated with a 1.6-folds higher risk of depression incidence compared to the second quartile (adjusted HR = 1.598, 95% CI = 1.086–2.351, data not shown).

We further examined the mediating effect of inflammatory markers and physical illnesses in the association between PM_2.5_ and depressive disorder (Table 4). The analysis revealed a negative average mediation effect of TNF-R1 on survival time (average mediated effect β = −0.961, 95% CI = −0.021–−3.163). Other inflammatory markers and physical illnesses did not show mediating effects.

**Table 4:** Mediating effect results between PM_2.5_ and survival time to newly developed depression.

**Mediators**	**Average direct effect**			**Average mediated effect**			**Total effect**		
		
	**β^a^**	**(95% CI)**	**p**	**β^a^**	**(95% CI)**	**p**	**β^a^**	**(95% CI)**	**p**
TNF-R1 (pg/mL)	−15.789	(−53.165–−0.776)	0.040	−0.961	(−3.163–−0.021)	0.040	−16.750	(−56.212–−1.320)	0.028
N/L ratio	−16.176	(−46.101–−0.861)	0.036	−0.292	(−1.333–0.237)	0.296	−16.468	(−46.369–−1.004)	0.036
hs-CRP (mg/dL)	−16.319	(−46.752–−1.287)	0.032	0.036	(−0.354–0.589)	0.820	−16.283	(−47.112–−1.229)	0.032
IGF-1 (ng/mL)	−16.151	(−46.815–−1.045)	0.032	−0.179	(−0.911–0.419)	0.548	−16.330	(−46.710–−1.268)	0.032
IL-6HS (pg/mL)	−16.149	(−46.638–−1.228)	0.032	−0.175	(−1.323–0.844)	0.672	−16.324	(−46.711–−1.290)	0.032
Heart disease	−16.733	(−52.028–−0.731)	0.044	−0.626	(−11.336–1.020)	0.168	−17.359	(−63.100–−2.072)	0.028
Cerebrovascular disease	−16.069	(−47.946–−0.624)	0.036	−0.099	(−5.106–1.047)	0.356	−16.168	(−53.757–−1.314)	0.032
Dementia	−15.575	(−44.637–−0.750)	0.036	−0.803	(−1.071–0.765)	0.556	−16.378	(−44.718–−0.733)	0.032

*Abbreviations:* TNF-R1 = tumor necrosis factor receptor 1; N/L ratio = neutrophil-to-lymphocyte ratio; hs-CRP = high sensitivity c-reactive protein; IGF-1 = insulin-like growth factor 1; IL-6HS = high sensitivity interleukin-6.

^a^The effects were modeled on survival time to depressive disorder incidence predicted by one-unit increase in inflammatory markers or the presence of physical illnesses. Biochemical marker variables were log-transformed for analysis. The models were adjusted for age, gender, body mass index, education level, marital status, work status, family income, cigarette smoking, exercise behaviors, metabolic equivalents, and the Charlson comorbidity index.

## 4. Discussion

This study observed that among air pollutants, PM_2.5_ was linearly associated with increased risk of developing depressive disorder in middle-aged and older adults. This association is partially mediated by elevated serum TNF-R1 level. Additionally, SO_2_ was non-linearly associated with depressive incidence.

### 4.1 Air and noise pollutants and depression

Meta-analysis studies revealed conflicting observations regarding risk of depression with long-term exposure to PM_2.5_ [3, 7, 9, 39]. This could result from different outcome measurements (e.g., depressive mood, depressive disorder, or antidepressant prescription), and different levels of air pollutants in the study locations. In this study, long-term exposure to PM_2.5_ is a risk for depressive disorder, suggesting a sustained effect of PM_2.5_ on mood. This association was not attributable to smoking or greater physical comorbidities in the depression group, as these confounders were adjusted for in the analysis. Only a few studies used newly onset diagnosis as the outcome, in which PM_2.5_ is associated with elevated risk [40]. Antidepressant use is associated with elevated risk but not diagnosis of depressive disorder, probably due to prescription of antidepressants but not to diagnosis by primary care physicians [41]. In Taiwan, patients approach psychiatrists directly, without the need for referral by a primary care physician. Taken together, these observations suggest that PM_2.5_ is associated with depressive disorder but may not be with transient depressive symptoms. Additionally, studies from the United States and Europe did not find an association between air pollutants and depressive symptoms [11, 42], where the mean level of PM_2.5_ was only one-quarter of that in this study. It has been suggested that long-term exposure to PM_2.5_ levels below 5 µg/m^3^ poses little threat to physical health [43]. Similar to the non-linear V-shaped concentration-response curve observed for non-accidental mortality in women [44], our findings showed the lowest risk of depressive incidence in the second quartile of PM_2.5_ (26.4–29.9 µg/m^3^). This level is much higher than the threshold for mortality risks, and it is possible that PM_2.5_ levels below this level do not cause significant biological or psychological effects associated with depression. Alternatively, factors associated with low levels of PM_2.5_, such as living in rural areas, may confound its association with depressive incidence [45].

We observed associations between CO, NO_2_, and SO_2_ and depression, with a non-linear pattern where significance was observed only in specific quartiles. Current evidence is strongest for PM_10_ for post-partum depression [39], and short-term but not long-term NO_2_ and SO_2_ for self-reported depressive symptoms [7, 9]. More studies are needed to confirm the mental health effects of these air pollutants and the impact of co-exposure to multiple pollutants. Furthermore, we did not observe an association between road traffic noise and depression. Nevertheless, a German cohort study reported an increased incidence of depressive symptoms among residents exposed to >55 dBA compared with those exposed to ≤55 dBA after adjusting for traffic proximity [46]. A cross-sectional study in Taiwan found an elevated prevalent risk of questionnaire-reported depression associated with exposure to 24-h road traffic noise after adjusting for PM_2.5_ [26]. The absence of associations in our study may be due to the use of long-term exposure data and clinically diagnosed depressive disorder, which are more conservative in examining the associations.

### 4.2 Mediating effect of inflammatory markers

Systemic inflammation and oxidative stress have been proposed as mechanisms linking PM_2.5_ exposure to depression [47]. PM induced inflammatory response through β2-adrenergic receptor signaling in the macrophages, which further activated the release of IL-6 and TNF-α [48]. TNF-alpha is a proinflammatory cytokine and activates its receptors, TNF-R1 and TNF-R2 [49], and TNF-R1 is mainly involved in the pro-inflammatory response [50]. Neuroinflammation has been found to play an important role in the pathophysiology of depression [51]. In animal studies, TNF-α promoted depression-like behavior by binding to TNF-R1 to activate astrocytes [52]. Our finding that PM_2.5_ is associated with new-onset depression through elevated TNF-R1 links with observations from previous literature. Furthermore, air pollutants have been associated with respiratory diseases [53], cardiovascular disease [54], cognitive decline, and stroke [55], which in turn increase the risk of depression. Although the depression group showed a higher rate of dementia than the control group at baseline, mediating effects of dementia were not observed in this study. Our findings suggest that PM_2.5_ may be associated with depression independent of other comorbidities. Notably, TNF-R1 was not elevated in the depressive group compared to control in Table 1, suggesting that factors such as age, sex, smoking status, and comorbidities may act as potential confounders in this association.

### 4.3 Strengths and limitations

This study is strengthened by using clinically diagnosed depressive disorder, and the application of land-use regression models to provide the temporal and spatial estimation of personal exposure levels for air pollutants and road traffic noise. There are limitations to this study. First, despite the high validity of depressive disorder incidence in the NHIRD, many individuals experiencing depression might not have sought medical help and thus were not identified as cases in this study [56]. As a result, the true incidence of depressive disorders was probably underestimated, and misclassifying cases to the group of control may have attenuated the association between pollutants and depressive incidence. Second, the exposure window of air pollutants was restricted to those 55 years or older. Early-year pollutant exposure was unknown in this study but may result in increased depressive incidence through a long-term exposition effect, such as gene methylation [3]. Instead, we adjusted for comorbidities that are related with air pollutants and occurred before depression. Nevertheless, there may also exist lag effects of air pollutants, that depressive incidence detected during the follow-up period was due to earlier exposure to pollutants. Third, the mediating effect of TNF-R1 in the association between PM_2.5_ exposure and depression does not imply causality. Other factors, such as genetic vulnerability [57], may also contribute to elevated TNF-R1 levels and the subsequent development of depression. Finally, more and more studies are showing an association between indoor air pollutants and mortality [58]. In this study, we did not assess the effect of household air pollution or exposure to environmental tobacco smoke.

## 5. Conclusions

PM_2.5_ is related to an increased risk of newly developed depressive disorder among middle-aged and older adults, with statistical models indicating that this association is partially mediated by the proinflammatory marker TNF-R1. Future studies are needed to clarify the temporal sequence of these associations and to determine whether modifying inflammatory status can attenuate the impact of PM_2.5_ on depression [53]. Late-onset depressive disorder, related to increased inflammation, has been observed to be associated with decreased survival in older adults [59]. Identifying vulnerable populations—such as individuals with preexisting inflammatory conditions or genetic susceptibility—may help inform targeted prevention strategies. Moreover, our findings highlight the importance of ecological efforts in air pollution control—including the implementation of stricter regulatory standards, public warning systems, improved public transportation infrastructure, and the expansion of green spaces—as complementary strategies for promoting mental health in older populations, alongside individual-level interventions.

## Abbreviations

BMI: Body Mass Index
CCI: Charlson Comorbidity Index
dBA: A-weighted decibel
hs-CRP: high sensitivity c-reactive protein
IGF-1: insulin-like growth factor 1
IL-6HS: high sensitivity interleukin-6
Lden: day-evening night noise level
METs: Metabolic equivalents
N/L ratio: neutrophil-to-lymphocyte ratio
TNF-R1: tumor necrosis factor receptor 1

## Supplementary information

**Additional file 1:** **Supplementary Table S1.** Annual exposure levels of predictive air pollutants and road traffic noise for the study population. **Supplementary Table S2.** Cox Proportional Hazards Model for Depressive Disorder Incidence. **Supplementary Table S3.** Demographic characteristics of study participants by levels of PM 2.5 (N = 2998). **Supplementary Figure S1.** Cox regression model with restricted cubic spline.

## Declarations

### Availability of data and material

The availability of data is subject to restrictions, and their usage in this study was permitted under license. The data that support the findings of this study are available from the National Health Research Institutes of Taiwan.
