# Supplementary material for: The increased risk of exposure to fine particulate matter for depression incidence is mediated by elevated TNF-R1: the Healthy Aging Longitudinal Study
Source: Environ Health Prev Med. 2025 Jun 27;30:49. doi: 10.1265/ehpm.25-00106 (PMC12206667; doi:10.1265/ehpm.25-00106)
Supplement: Supplementary file 1 — Additional file 1: Supplementary Table S1. Annual exposure levels of predictive air pollutants and road traffic noise for the study population. Supplementary Table S2. Cox Proportional Hazards Model for Depressive Disorder Incidence. Supplementary Table S3. Demographic characteristics of study participants by levels of PM 2.5 (N = 2998). Supplementary Figure S1. Cox regression model with restricted cubic spline. [file ehpm-30-049-s001.docx]

**Supplementary Materials**

Supplementary Table S1. Annual exposure levels of predictive air pollutants and road traffic noise for the study population.

| Variables | Mean | SD | Median | Range | IQR |
| --- | --- | --- | --- | --- | --- |
| PM_2.5_ (μg/m^3^) | 31.02 | 7.39 | 29.87 | 9.47–68.9 | 8.53 |
| PM_10_ (μg/m^3^) | 52.94 | 11.88 | 51.11 | 21.39–101.58 | 15.81 |
| CO (ppb) | 0.58 | 0.18 | 0.59 | 0.01–1.34 | 0.16 |
| NO_2_ (ppb) | 10.97 | 3.92 | 10.52 | 0.03–20.7 | 4.60 |
| O_3_ (ppb) | 28.92 | 7.79 | 28.26 | 2.59–61.12 | 10.24 |
| SO_2_ (ppb) | 4.19 | 1.4 | 3.90 | 0.91–11.55 | 1.69 |
| L_den_ (dBA) | 60.67 | 6.08 | 61.25 | 36.21–77.51 | 5.28 |

*Abbreviations:* dBA = A-weighted decibel; SD = standard deviation; IQR = interquartile range.

Supplementary Table S2. Cox Proportional Hazards Model for Depressive Disorder Incidence.

| Pollutants (quartile) | Crude model^a^ | |  | Adjusted model^a^ | |
| --- | --- | --- | --- | --- | --- |
|  | HR (95% CI) | p |  | HR (95% CI) | p |
| PM_2.5_ (μg/m^3^) |  |  |  |  |  |
| Lowest quartile | (Reference) |  |  | (Reference) |  |
| 2^nd^ quartile | 0.813(0.545–1.214) | 0.312 |  | 0.779(0.521–1.166) | 0.225 |
| 3^rd^ quartile | 0.831(0.558–1.238) | 0.364 |  | 0.820(0.548–1.225) | 0.332 |
| Highest quartile | 1.266(0.885–1.811) | 0.197 |  | 1.245(0.868–1.787) | 0.234 |
| PM_10_ (μg/m^3^) |  |  |  |  |  |
| Lowest quartile | (Reference) |  |  | (Reference) |  |
| 2^nd^ quartile | 0.832(0.554–1.249) | 0.375 |  | 0.812(0.541–1.220) | 0.317 |
| 3^rd^ quartile | 0.994(0.676–1.461) | 0.976 |  | 0.972(0.658–1.436) | 0.888 |
| Highest quartile | 1.132(0.781–1.642) | 0.513 |  | 1.136(0.781–1.651) | 0.505 |
| CO (ppm) |  |  |  |  |  |
| Lowest quartile | (Reference) |  |  | (Reference) |  |
| 2^nd^ quartile | 1.444(1.000–2.085) | 0.050 |  | 1.480(1.019–2.149) | 0.040 |
| 3^rd^ quartile | 0.910(0.602–1.375) | 0.654 |  | 0.910(0.597–1.388) | 0.662 |
| Highest quartile | 1.175(0.790–1.748) | 0.427 |  | 1.051(0.693–1.594) | 0.816 |
| NO_2_ (ppb) |  |  |  |  |  |
| Lowest quartile | (Reference) |  |  | (Reference) |  |
| 2^nd^ quartile | 0.590(0.387–0.902) | 0.015 |  | 0.597(0.389–0.915) | 0.018 |
| 3^rd^ quartile | 0.943(0.648–1.372) | 0.759 |  | 0.899(0.610–1.326) | 0.593 |
| Highest quartile | 1.209(0.848–1.725) | 0.294 |  | 1.111(0.767–1.607) | 0.579 |
| O_3_ (ppb) |  |  |  |  |  |
| Lowest quartile | (Reference) |  |  | (Reference) |  |
| 2^nd^ quartile | 1.123(0.770–1.639) | 0.547 |  | 1.162(0.796–1.697) | 0.436 |
| 3^rd^ quartile | 0.947(0.643–1.395) | 0.782 |  | 1.088(0.730–1.620) | 0.680 |
| Highest quartile | 0.847(0.570–1.259) | 0.413 |  | 0.949(0.628–1.434) | 0.804 |
| SO_2_ (ppb) |  |  |  |  |  |
| Lowest quartile | (Reference) |  |  | (Reference) |  |
| 2^nd^ quartile | 1.103(0.724–1.681) | 0.649 |  | 1.126(0.738–1.718) | 0.583 |
| 3^rd^ quartile | 1.621(1.100–2.389) | 0.015 |  | 1.618(1.096–2.390) | 0.016 |
| Highest quartile | 1.317(0.878–1.977) | 0.184 |  | 1.318(0.875–1.985) | 0.186 |
| L_den_ (dBA) |  |  |  |  |  |
| Lowest quartile | (Reference) |  |  | (Reference) |  |
| 2^nd^ quartile | 0.889(0.620-1.275) | 0.522 |  | 0.865(0.601–1.245) | 0.435 |
| 3^rd^ quartile | 0.727(0.493-1.071) | 0.107 |  | 0.689(0.463–1.024) | 0.065 |
| Highest quartile | 0.762(0.522-1.11) | 0.156 |  | 0.770(0.526–1.128) | 0.179 |

*Abbreviations:* dBA = A-weighted decibel;

^a^ The hazard ratio (HR) and 95% confidence interval (CI) for the occurrence of depressive disorders associated with pollutants categorized into quartiles, with the lowest quartile serving as the reference group (N = 2,998). Crude models were univariate Cox proportional hazard models, showing the estimated increased hazard with an IQR increase in the pollutants. Adjusted models were controlled for age, gender, body mass index, education level, marital status, work status, family income, cigarette smoking, exercise behaviors, metabolic equivalents, and Charlson comorbidity index.

Supplementary Table S3. Demographic characteristics of study participants by levels of PM 2.5 (N = 2998).

|  | Lowest quartile  (9.47–26.43 μg/m^3^) | 2^nd^  quartile  (26.43–29.87 μg/m^3^) | 3^rd^  quartile  (29.87–34.96 μg/m^3^) | Highest quartile  (34.96–68.90 μg/m^3^) |  |
| --- | --- | --- | --- | --- | --- |
|  | (n = 750) | (n = 750) | (n = 749) | (n = 749) |  |
| **Characteristic** | **N (%)** | **N (%)** | **N (%)** | **N (%)** | ***p^a^*** |
| Gender (male) | 370 (49.33) | 348 (46.40) | 375 (50.07) | 373 (49.80) | 0.461 |
| Education (primary or lower) | 368 (49.07) | 346 (46.13) | 436 (58.21) | 401 (53.54) | <0.001 |
| Marital status (married) | 573 (76.40) | 594 (79.20) | 572 (76.37) | 556 (74.23) | 0.158 |
| Work status (working) | 189 (25.20) | 229 (30.53) | 234 (31.24) | 198 (26.44) | 0.020 |
| Family income |  |  |  |  | <0.001 |
| High | 137 (18.27) | 136 (18.13) | 117 (15.62) | 94 (12.55) |  |
| Low | 277 (36.93) | 256 (34.13) | 315 (42.06) | 373 (49.80) |  |
| Missing | 336 (44.80) | 358 (47.73) | 317 (42.32) | 282 (37.65) |  |
| Cigarette smoking (yes) | 87 (11.60) | 99 (13.20) | 91 (12.15) | 88 (11.75) | 0.778 |
| Regular exercise (yes) | 579 (77.20) | 556 (74.13) | 526 (70.23) | 550 (73.43) | 0.023 |
| CCI |  |  |  |  | 0.075 |
| 0 | 276 (36.80) | 253 (33.73) | 232 (30.97) | 233 (31.11) |  |
| 1 | 217 (28.93) | 228 (30.40) | 219 (29.24) | 211 (28.17) |  |
| ≥ 2 | 257 (34.27) | 269 (35.87) | 298 (39.79) | 305 (40.72) |  |
| Heart disease | 120 (16.00) | 96 (12.80) | 106 (14.15) | 99 (13.22) | 0.287 |
| Cerebrovascular disease | 158 (21.07) | 141 (18.80) | 121 (16.15) | 133 (17.76) | 0.096 |
| Dementia | 83 (11.07) | 66 (8.80) | 67 (8.95) | 99 (13.22) | 0.016 |
| Depressive incidence during follow-up | 54 (7.20) | 43 (5.73) | 44 (5.87) | 68 (9.08) | 0.039 |
|  | **Mean (SD)** | **Mean (SD)** | **Mean (SD)** | **Mean (SD)** | ***p^a^*** |
| Age (year) | 68.81 (7.69) | 67.8 (7.41) | 68.41 (7.41) | 68.7 (8.18) | 0.098 |
| BMI (kg/m^2^) | 24.88 (3.45) | 24.48 (3.37) | 24.62 (3.35) | 24.79 (3.46) | 0.244 |
| METs | 2018.52 (2969.59) | 1802.34 (2676.38) | 1732.8 (2624.24) | 1802.64 (2823.52) | <0.001 |

*Abbreviations:* METs = Metabolic equivalents; BMI = Body Mass Index; CCI = Charlson Comorbidity Index; SD = standard deviation.

^a^P values comparing the four groups were calculated using the Kruskal-Wallis H test and chi-square tests.


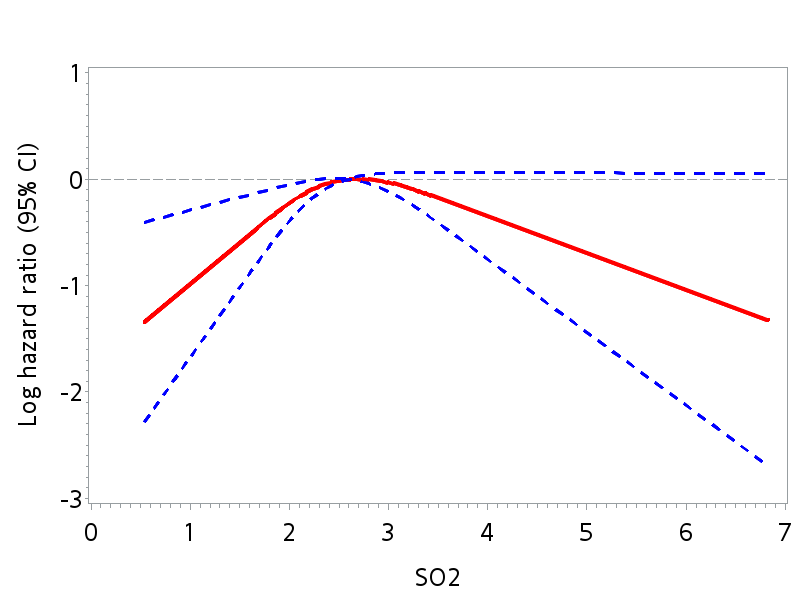

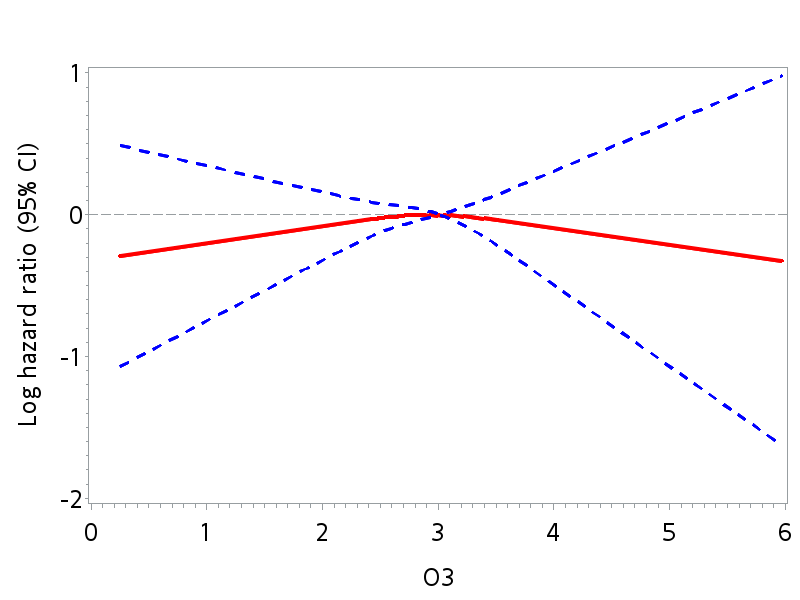

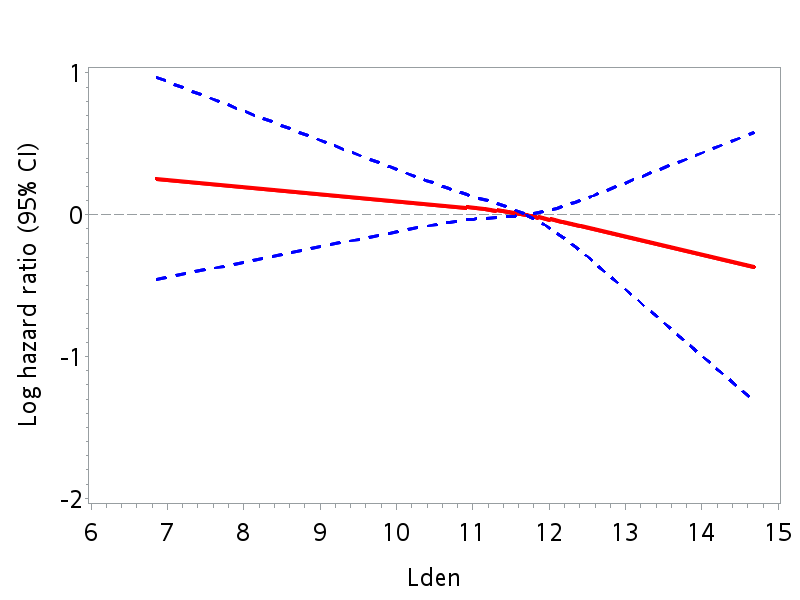

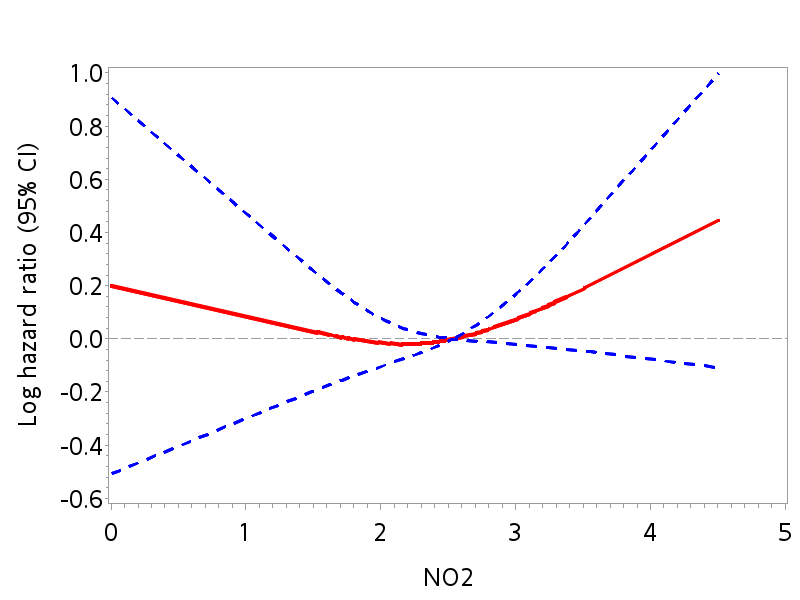
Supplementary Figure S1. Cox regression model with restricted cubic spline.


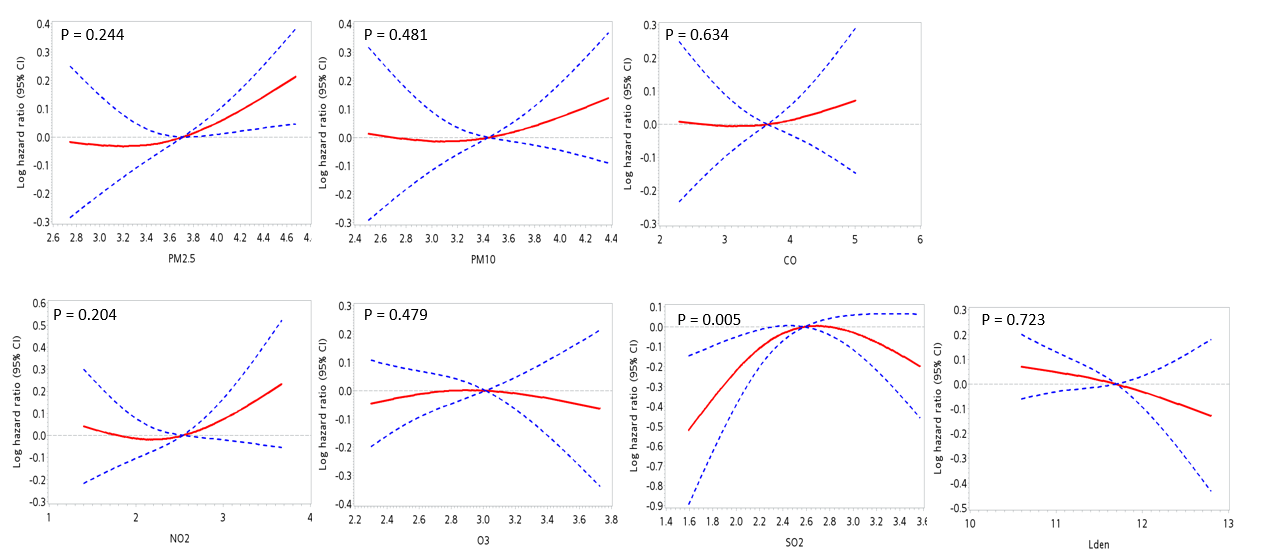
 The plots illustrate the association curve between the 10^th^ and 90^th^ percentile knots, with p-values for non-linearity displayed. The unit for the X-axis is the interquartile range of pollutants, while the Y-axis unit is the log hazard ratio. The red lines indicate the estimated associations, and the blue lines represent the 95% confidence intervals. The models were adjusted for age, gender, body mass index, education level, marital status, work status, family income, cigarette smoking, exercise behaviors, metabolic equivalents, and the Charlson Comorbidity Index.
